# New insights in understanding biliary atresia from the perspectives on maternal microchimerism

**DOI:** 10.3389/fped.2022.1007987

**Published:** 2022-09-23

**Authors:** Toshihiro Muraji, Ryuta Masuya, Toshio Harumatsu, Takafumi Kawano, Mitsuru Muto, Satoshi Ieiri

**Affiliations:** ^1^Department of Pediatric Surgery, Research Field in Medicine and Health Sciences, Medical and Dental Sciences Area, Research and Education Assembly, Kagoshima University, Kagoshima, Japan; ^2^Division of the Gastrointestinal, Endocrine and Pediatric Surgery, Department of Surgery, Faculty of Medicine, University of Miyazaki, Miyazaki, Japan

**Keywords:** biliary atresia, GVHD, maternal microchimerism, Treg, IL-6

## Abstract

Biliary atresia (BA) is a fibroinflammatory cholangiopathy and portal venopathy. It is of unknown etiology and is associated with systemic immune dysregulation, in which the first insult begins before birth. Maternal microchimerism is a naturally occurring phenomenon during fetal life in which maternal alloantigens promote the development of tolerogenic fetal regulatory T-cells *in utero*. However, maternal cells may alter the fetus’s response to self-antigens and trigger an autoimmune response under certain histocompatibility combinations between the mother and the fetus. A recent report on a set of dizygotic discordant twins with BA, one of whose placentae showed villitis of unknown etiology, implies a certain immune-mediated conflict between the fetus with BA and the mother. Maternal chimeric cells persist postnatally for various time spans and can cause cholangitis, which ultimately leads to liver failure. In contrast, patients who eliminate maternal chimeric cells may retain their liver function.

## Introduction

Biliary atresia (BA) is an immune-mediated obstructive cholangiopathy of unknown etiology. The complexity of immune dysregulation underlying the disease makes it difficult to treat, and although liver transplantation is an effective last resort, preservation of native liver function is a more desirable clinical outcome. According to the Japanese Biliary Atresia Registry (1), 43% and 14% of patients with BA initially pass normal color meconium and yellow-colored stool at the time of Kasai hepatic portoenterostomy (KPE), respectively. Furthermore, most patients present with persistent jaundice within 1 month of birth. This clinical presentation makes early detection of BA difficult. KPE is essential for the drainage of bile into the intestine but is not always sufficient. Even with successful KPE, 40% of patients develop episodes of cholangitis, which often lead to progressive fibrosis, necessitating liver transplantation by the age of 20 years. Ongoing immune dysregulation must be addressed directly to promote native liver survival by first elucidating the underlying pathogenic mechanisms of BA.

This review aimed to summarize recent clinical and immunological findings in BA and to discuss the maternal microchimerism (MMc) hypothesis as the etiopathogenesis.

## Onset of biliary atresia

Defining the onset of BA would provide clues regarding its etiopathogenesis. In 1996, Yamagiwa et al. (2) reported that direct bilirubin levels were significantly higher within 1 week after birth in patients with BA than in age-matched controls (4.6 ± 2.6 vs. 0.7 ± 0.3 mg/dl). Recently, Harpavat et al. (3) reported similar findings of high direct bilirubin levels even at birth, proposing that BA starts before birth. This concept is compatible with previous clinical observations, such as the earliest antenatally diagnosed cystic type of BA (type I), which was at 16 weeks of gestation (4), and the absence of gamma-glutamyl transpeptidase in amniotic fluid, which is typically present during 18–19 weeks of gestation (5). These findings suggest that obstructive cholangiopathy had already begun before the 15th week of gestation, that is consistent to the histopathological changes in BA, the ductal plate malformation theory (6), proposed by Desmet in 1992, states that interlobular bile ducts retain their morphology at 8–12 weeks of gestation before remodeling of the ductal plate in 30% of patients with BA.

## Histopathological and immunological features of biliary atresia

### Histopathological features

Histopathological features of the liver in the presence of BA include bile ductular proliferation and expanded portal areas, with infiltration of polymorphonuclear leukocytes, macrophages, and occasional mononuclear lymphocytes in the biliary epithelium. It also includes hepatocyte ballooning, multinucleated giant cells, and apoptosis. The giant cell transformation of hepatocytes seems to occur early in the postnatal period and subsides gradually over several months, and is a short-term prognostic indicator of BA (7).

Strong expression of costimulatory factor (B7) was shown not only in bile ductules but also on the surfaces of Kupffer and dendritic cells (DC) and in the hepatocytic cytoplasm. In particular, there was a high expression of B7-1 in the vascular and sinusoidal endothelial cells in patients with portal hypertension (8). Costimulatory factors are ligands on antigen-loaded macrophages. The dendritic cells migrate into the endothelial or epithelial lining, which activates naïve T cells through their costimulatory receptors, CD28. Small proliferating portal vein (PV) branches were identified as a histopathological feature of BA in liver biopsy specimens sampled at the time of KPE (9). We subsequently demonstrated that a decreased number of PV branches with increased microvascular proliferation in the portal area is associated with better long-term clinical outcomes (10). Therefore, portal hypertension in BA may not only be secondary to progressive portal fibrosis due to cholestasis but also part of the primary dysgenesis of the PV system.

In hepatic graft-versus-host disease (GVHD), giant cell transformation of hepatocytes, portal vein vasculitis, and vanishing bile ducts are distinct histopathological features (11). Additionally, C4d expression is increased in the PV and hepatic sinusoids of patients with histologically confirmed chronic GVHD (12) and is also a characteristic clinical feature of the liver in BA (13), suggesting that the PV endothelium and biliary epithelial cells are targets of B cell immunity in GVHD and BA.

### Immunological features are similar to those with hepatic graft-versus-host disease as a complication of hematopoietic stem cell transplantation

Interleukin (IL)-6 is a proinflammatory cytokine that plays a major role in acute GVHD after hematopoietic stem cell transplantation (HSCT) conditioning and involves the gastrointestinal tract and liver (14). It is produced by antigen-presenting cells (DCs and macrophages) upon antigen stimulation and promotes B cell differentiation. This, in turn, induces IL-8 (15) and TNF-α production and enhances soluble cellular and vascular adhesion molecules (sICAM-1 and sVCAM-1), allowing interaction with HLA class II molecules for antigen presentation and HLA class I molecules for cytotoxic T cell responses. IL-6 also stimulates CD4 + T cells to inhibit T regulatory (Treg) cell function and promote T helper 17 (Th17) differentiation (16).

In BA, sICAM was reported as the earliest marker to show a significant difference between outcome groups at one-month post-KPE (17). Th17 cell infiltration is greater in the liver with BA and is related to the surgical outcome (18, 19). Contrastingly, while the number of Th-17 cells is elevated, Treg cells are decreased among the peripheral blood mononuclear cells, and there is also increased expression of IL-6 (19). CD25 is a high-affinity IL-2 receptor in Treg cells and is necessary for their proliferation. In BA, there are reports of elevated IL-2 mRNA expression in liver biopsy specimens with increased infiltration of CD8 + T cells (20) and significantly higher plasma IL-2 levels in the liver transplant group at 1 year (17). This could be explained by the decreased uptake of IL-2 by inducible Tregs in the secondary lymphoid organs.

Taken together, IL-6 is the key cytokine for immune dysregulation in patients with BA and is triggered by maternal chimeric effector cells as the first insult under an immature immune condition, as is the case with GVHD caused by donor effector cells under conditioning.

## Maternal microchimerism hypothesis

Maternal microchimerism is a phenomenon in which maternal cells cross the placenta and are engrafted into the human fetal tissues *in utero*. Since the early 2000s, MMc has been purported to play a role in the pathogenesis of juvenile idiopathic inflammatory myopathies (21), neonatal lupus syndrome (22), and type 1 diabetes mellitus (23). In BA, MMc was first detected in 2004 (24), implying the possibility of the same mechanism being involved in the above-mentioned pediatric autoimmune diseases. Following initial quantitative demonstrations of maternal cells in the BA liver, qualitative analysis of maternal chimeric cells showed that their phenotypes included CD8 +, CD45 + T cells, and cytokeratin + differentiated biliary epithelial cells (25). These findings suggest that maternal chimeric cells include differentiated effector T cells as well as hematopoietic and mesenchymal stem cells (26).

A recent report (27) on a set of dizygotic discordant twins with BA, one of whose dichorionic-diamniotic placentae was smaller and associated with segmental focuses of the villitis of unknown etiology (VUE), implies a certain immune-mediated conflict between the fetus with BA and the mother. A mixed lymphocyte reaction in this case disclosed that the mother became sensitized by the patient’s antigens. Etiology of VUE is now known to be the consequence of immune interactions between sensitized macrophages of fetal origin and CD8 + cells of maternal origin (28). The maternofetal alloreactivity can only occur in their selective combination of major and minor histocompatibility antigens, which is likely to influence MMc (29). Ethnic variation in the incidence of BA is a unique epidemiological feature of BA that supports this hypothesis (30).

### Atrophic left lateral segment

**FIGURE 1:**
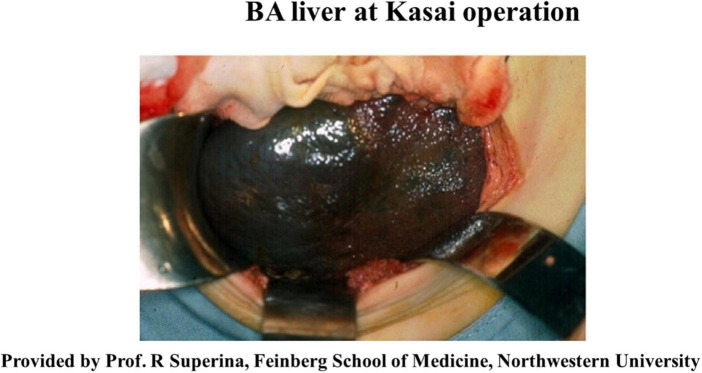
The liver exteriorized out of the upper abdominal cavity at the time of Kasai portoenterostomy. Note the nodularity confined to the surface of a well-demarcated left lateral segment. Courtesy of Dr. Riccardo Superina, Feinberg School of Medicine, Northwestern University.

**FIGURE 2:**
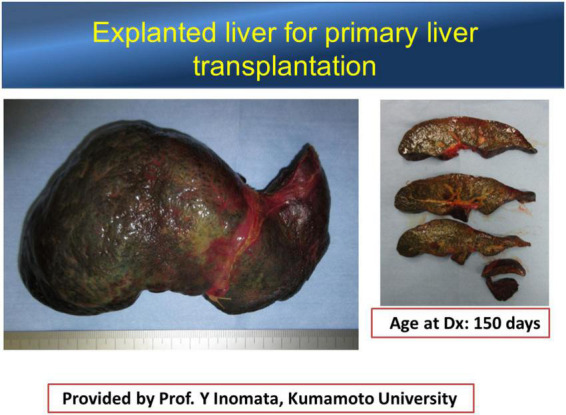
An explanted liver of the patient without precedent Kasai portoenterostomy. The left lateral segment is atrophic, implying that atrophy is not because of the insufficient bile drainage. Courtesy of Dr. Yukihiro Inomata, Kumamoto University.

The left lateral segment (LLS) of the BA liver is a unique segment associated with more severe inflammatory reactions and fibrosis than other segments (31, 32), while there is a case with remarkable fibrosis in the LLS observed at the time of KPE (Figure 1) and an explanted liver for liver transplant without KPE (Figure 2). The causative mechanism of segmental fibrosis can be explained anatomically (33). As noted above, obstructive cholangiopathy has been shown to begin before the 15th week of gestation. However, the ductal plate malformation theory proposes that it begins even as early as 8–12 weeks of gestation. Embryologically, the right umbilical vein diminishes around the 7th week of gestation. The left umbilical vein runs into the left branch of the portal vein as a single umbilical vein containing whole blood flow from the placenta. Therefore, maternal chimeric cells are likely engrafted in and target the LLS during the first hit.

However, Tamaoka et al. (34) recently reported that maternal cells were scant or absent in the LLS sampled from explanted livers. We also observed that maternal chimeric cells in LLS were fewer in number than in the right lobe, even when sampled at the time of KPE (35). This paradox conceivably leads to the concept that autoimmune interactions are the responsible mechanism postnatally toward the later stage of this disease, as in chronic GVHD. Leveque et al. (36) demonstrated in animal models that the ratio of T effectors/Treg was significantly increased in the offspring of alloreactive maternal mice, which may alter the offspring’s response to self-antigens and potentially trigger an autoimmune response later in life. Overexpression of loaded DC and macrophages is persistent in the LLS with engrafted maternal chimeric cells, leading to increased availability of targets for the subsequent insults as result of autoimmunity. On the other hand, it is also conceivable that if maternal chimeric cells are eliminated, intrahepatic inflammatory processes may diminish, and liver function is improved, allowing patients to survive without the need for liver transplantation.

### Postnatal transition of stool color

Lithocholic acid (LCA) metabolites in the gut control host immune responses by inhibiting Th17 cell differentiation by directly binding to their key transcription factor, retinoid-related orphan receptor (ROR) γt. In particular, isoalloLCA enhances Treg differentiation through the production of mitochondrial reactive oxygen species, leading to increased FoxP3 expression. (37). Mucosa-associated lymphoid tissues in the gut are one of the sites that are exposed to additional antigen input from breastfeeding, including maternal antigens.

In patients with BA, however, bile acid is obviously decreased or absent in the gastrointestinal tract after birth. Therefore, Treg cell development may be affected as Th-17 effector cells are induced in the lymph nodes of the porta hepatis in the presence of IL-6 produced by sensitized macrophages and loaded DCs in the liver. There is probably some degree of patency of the intrahepatic bile ducts and the extrahepatic bile duct in the fibrotic remnant at birth. However, with the maternal antigen-specific autoimmunity reactivated in the liver postnatally, further inflammatory obliteration of the intra-and extrahepatic bile ducts would progress. This is why stool is initially cholic but gradually becomes acholic after birth.

### Two-hit model for etiopathogenesis: Perinatal transition from allo-reactive to auto-reactive immune dysregulation in patients with biliary atresia

**FIGURE 3:**
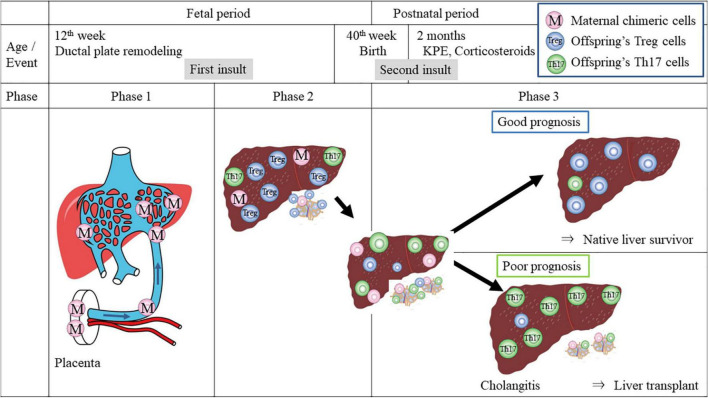
Hypothetical model of T-cell dynamic imbalance in pathogenesis of BA. Phase 1 is the first insult: maternal effector T cells which transfer *via* the umbilical vein largely into the left lateral segment of the fetal liver may damage the developing ductal plate and the portal vein structure during the first trimester. Maternal chimeric cells persist in the liver and the secondary lymphoid tissues. Phase 2 describes the remainder of gestation. Fetal Tregs become dominant in peripheral lymphoid organs and in the liver, suppressing autoreactive Th17 cells and allowing pregnancy stability. Phase 3 is the postnatal period. At birth, due to lack of lithocholic acid in the gut of patients with BA, Th17 differentiation and Treg cell deficit may occur and the balance turns into chronic GVHD-type autoimmunity under activated inflammatory cytokines such as IL-6 and IL-8. Even after bile drainage is achieved with KPE and immune imbalance may be reversed with corticosteroids, persistent maternal chimeric cells may continue to skew immunity toward autoreactivity, causing a flare-up of inflammation, leading to frequent episodes of cholangitis and ongoing liver fibrosis. In contrast, with the elimination of maternal chimeric cells, the patients may have a chance to become anicteric with normal liver function and achieve long-term survival.

Acute GVHD associated with HSCT is now known to be initially triggered by alloreactive donor T cells (hit 1), which damage peripheral tissues and affect primary and secondary lymphoid organs. Subsequent transition to chronic GVHD involves the emergence of autoimmunity (hit 2) (Figure 3) (16), although the underlying mechanisms driving this process are unclear, either centrally in the thymus or peripherally in the lymph nodes.

In MMc, Leveque (38) hypothesized that maternal microchimeric T cells acquired during pregnancy may be the potential key actors that interfere with developing fetal immune system and therefore may modify Treg development predisposing the offspring to autoimmunity. In BA, maldevelopment of the bile duct may be caused by alloreactive maternal cells in GVHD-like “allo-auto”-insult (hit 1). Maternal chimeric cells persist in the secondary lymphoid tissues, including lymph nodes and spleen and may skew immunity toward autoreactive immune dysregulation (hit 2). Lymph nodes of the porta hepatis are often swollen at the time of KPE, including maternal chimeric cells (39). Sakamoto et al. (40) reported that the ratio of memory T reg (CD4 + /CD25 + /FoxP3^+^/CD45RA^–^) to total Treg cells in the lymph nodes of porta hepatis increases than that in the peripheral blood in patients with BA, implying memory Tregs tend to be accumulated in the swollen lymph nodes.

Regarding the other secondary lymphoid tissue, maternal microchimeric stem cells may reside in the spleen. Takahashi et al. (41) reported that splenectomy for liver-transplant candidates improved liver function, avoiding liver transplantation in 50% of patients during a mean follow-up period of 56 months. This observation may support the theory that maternal memory T cells may retain in the spleen and affect the Th17/Treg balance. Splenectomy may eradicate these maternal memory T cells.

### Three phases of the clinical course

A clear understanding of the complex cytokine-driven cellular networks in chronic GVHD after HSCT has clarified its pathogenic dysregulation. A three-phase model (Figure 3) for its initiation and development (42) includes early inflammation and tissue injury (phase 1), chronic inflammation and dysregulated immunity (phase 2), and aberrant tissue repair accompanied often by fibrosis (phase 3). This three-phase concept was applied here to the immunobiology involved in BA.

Phase 1 occurs in the first trimester when the maternal effector T cells inflict the first insult by targeting the fetal bile duct and PV.

Phase 2 occurs between 12 and 20 gestational weeks. The Tregs in the peripheral lymphoid organs increase by ∼15–20% of the total CD4 + T cells, possibly by differentiation of naïve fetal T cells into Tregs in response to maternal antigen stimulation (43), which conceivably suppress inflammation, allowing the rest of gestation to be stable. However, maternofetal immune interactions in the placental interface might not be silent. They may cause villitis of unknown etiology secondarily.

Phase 3 takes place in the postnatal period. At birth, due to the lack of lithocholic acid in the gut of patients with BA (33), Th17 differentiation and Treg cell deficit may be aggravated, leading to autoimmunity. In particular, after KPE, a flare-up of the intrahepatic bile ducts occurs under the influence of activated inflammatory cytokines such as IFN-gamma, IL-6, or TNF-α. When KPE is successful, there is drainage of bile into the gut, and the inflammatory environment is controlled with corticosteroids, allowing patients to have a chance to survive without the need for liver transplantation and achieve long-term survival with the elimination of maternal chimeric cells. However, if maternal chimeric cells persist even if bile is drained, a flare-up of inflammation can cause frequent episodes of cholangitis, both suppurative and non-suppurative, and ongoing liver fibrosis ensues.

## Conclusion

GVHD-type insults by maternal effector T cells targeting the bile duct epithelium and PV endothelium may trigger BA, and circulating maternal chimeric lymphocytes are postnatally persistent and can cause chronic GVHD with Treg deficiency as the second insult. Further investigations are warranted to define the causal effect of MMc and Treg deficits.

## Data availability statement

The original contributions presented in this study are included in the article/supplementary material, further inquiries can be directed to the corresponding author.

## Author contributions

TM conceptualized the hypotheses and wrote the manuscript. RM and TH collected the clinical data and literature. TK, MM, and SI reviewed and edited the manuscript. All authors have read and approved the manuscript.
